# Sex differences in senescence: the role of intra-sexual competition in early adulthood

**DOI:** 10.1098/rspb.2015.1086

**Published:** 2015-07-22

**Authors:** Christopher Beirne, Richard Delahay, Andrew Young

**Affiliations:** 1 Centre for Ecology and Conservation, School of Biosciences, University of Exeter, Penryn Campus, Cornwall TR10 9EZ, UK; 2 National Wildlife Management Centre, Animal Health and Veterinary Laboratories Agency (AHVLA), Woodchester Park, Gloucestershire GL10 3UJ, UK

**Keywords:** senescence, ageing, mammal, wild population, individual variation, disposable soma

## Abstract

Males and females frequently differ in their rates of ageing, but the origins of these differences are poorly understood. Sex differences in senescence have been hypothesized to arise, because investment in intra-sexual reproductive competition entails costs to somatic maintenance, leaving the sex that experiences stronger reproductive competition showing higher rates of senescence. However, evidence that sex differences in senescence are attributable to downstream effects of the intensity of intra-sexual reproductive competition experienced during the lifetime remains elusive. Here, we show using a 35 year study of wild European badgers (*Meles meles*), that (i) males show higher body mass senescence rates than females and (ii) this sex difference is largely attributable to sex-specific downstream effects of the intensity of intra-sexual competition experienced during early adulthood*.* Our findings provide rare support for the view that somatic maintenance costs arising from intra-sexual competition can cause both individual variation and sex differences in senescence.

## 1. Introduction

Senescence, defined as within-individual physiological deterioration with age, has been detected in a wide variety of natural populations, but the causes of the often marked individual variation in senescence rates remain poorly understood [1]. A major source of variation in senescence rates is sex; females frequently have longer lifespans than males in humans and other mammals [2–4], and recent evidence has highlighted associated sex differences in senescence rates in a range of fitness-related traits, including reproductive success [5,6], body mass [7] and telomere length [8]. While sex differences in senescence rates are now comparatively well documented, the mechanisms that generate such differences remain poorly understood [9].

It has been hypothesized that sex differences in senescence rates arise from the differing strength of intra-sexual reproductive competition experienced by males and females [10,11]. Evolutionary approaches to this hypothesis have focused principally on the implications of the sex difference in mean mortality rates that often accompanies sex differences in the intensity of intra-sexual competition [10]. The sex that experiences stronger intra-sexual reproductive competition often shows higher mean mortality rates, which may thereby differentially weaken the force of selection against deleterious mutations or antagonistically pleiotropic genes acting in late life in that sex [12], leading to the evolution of faster senescence rates and shorter lifespans [10]. Evidence that males in polygynous species frequently do senesce faster than females [4,5] and that species with stronger degrees of polygyny show more marked sex differences in reproductive lifespan [3] is therefore broadly consistent with this view. However, support for a central role for sex differences in mortality rates is far from universal [6,13], highlighting the likely importance of other mechanisms.

From a mechanistic perspective, it has also been hypothesized that individuals may suffer resource allocation trade-offs between the expression of competitive morphologies and behaviour and their simultaneous allocation to somatic maintenance, which might thereby lead to steeper senescent declines later in life in the sex that experiences stronger intra-sexual competition [11,14]. This perspective highlights the potential limitations of focusing solely on the implications of mortality rates, and predicts instead a direct effect of the intensity of intra-sexual competition experienced during early adulthood on both individual variation in senescence rates and the extent of any sex difference observed in senescence rates [1]. However, evidence that variation in the intensity of intra-sexual competition experienced predicts individual variation in senescence rates is rare and derives solely from studies of a single sex [15,16], leaving it unclear to what extent sex differences in senescence are also attributable to this same mechanism. Specifically, if somatic maintenance costs arising from intra-sexual competition do contribute to sex differences in senescence rates, one would predict (i) higher rates of senescence in the sex that experiences stronger intra-sexual reproductive competition and (ii) that the magnitude of any sex difference in senescence rate observed should be predicted by the intensity of intra-sexual competition experienced during early adulthood by members of one or both sexes.

Here, we test both predictions using 35 years of longitudinal data from a long-term field study of the European badger (*Meles meles*). We use 11 782 captures of 2074 known-age individuals to directly compare the age-related body mass trajectories of males and females, and then investigate whether the observed sex difference in body mass senescence rates is attributable in part to downstream effects of intra-sexual competition experienced during early adulthood. Variation in body mass explains considerable variation in both survival and reproductive success in mammals [17] and is recognized as a useful phenotypic indicator of somatic state [18], but the causes of variation in late-life declines in body mass have received remarkably little attention [7,19,20]. In the European badger, body mass is an important trait contributing to reproductive success [21,22] and survival [23]. Senescent declines in body mass might therefore underpin recent observations in this species of both reproductive senescence [24] and sex-specific survival senescence ([25]; albeit weaker than has been documented in some other species). Male European badgers contest mating opportunities both within and among social groups, leading to high rates of extra-group paternity (up to 50% of offspring; [26,27]) and elevated variance in reproductive success among males [24,26,28]. In high-density populations, there is also evidence of reproductive skew among females within a group [21,28]. While the extent to which the observed patterns of parentage are a product of intra-sexual competition is not clear [27], a higher incidence of bite-wounding and mortality among males coupled with male-biased sexual size dimorphism in this species, strongly suggests that overt intra-sexual reproductive competition is more intense among males than females [29,30].

Specifically, we test two key predictions: if somatic maintenance costs arising from intra-sexual reproductive competition play a key role in generating sex differences in senescence rates: (i) male European badgers should show a faster rate of late-life decline in body mass than females and (ii) this sex difference in senescence rate should be attributable in part to downstream effects of the intensity of intra-sexual reproductive competition experienced in early adulthood. To address the second prediction, we test for sex-specific downstream effects of three proxies for the strength of reproductive competition experienced during an individual's early adulthood (the local densities of adult males and adult females, and the local adult sex ratio) on their rate of body mass senescence later in life, while controlling for any effect of ‘current’ badger density on their body mass during the senescent period. We also address the possibility of downstream effects on senescence rates of generalized foraging competition in early adulthood (rather than intra-sexual reproductive competition *per se*; following [31]), by examining the influence of the total badger density experienced during early adulthood. We apply a linear mixed-model approach throughout, which allows us to examine changes in body mass with chronological age while controlling for selective disappearance and terminal effects that might otherwise obscure or exaggerate patterns of senescence [32].

## 2. Material and methods

We use data from the long-term Woodchester Park field study (Gloucestershire, UK), where the resident high-density European badger population has been continuously monitored since the 1970s [33]. Each year, the boundaries of all social group territories in the 11 km^2^ site were approximated by bait marking in spring [33], and badgers trapped at all active setts for two nights, four times per year. Badgers were anaesthetized and identified through a unique tattoo administered at first capture. The location, sex, mass (to nearest 100 g) and age class (juveniles < 1 year, adult ≥ 1) were recorded for each individual. Captures of individuals of unknown age (those not identified as juveniles at first capture) or unrecorded mass, sex, social group or sett location were excluded from our body mass analyses. Bovine tuberculosis (bTB) infection status was determined for all individuals at every capture [34]. In a detailed investigation of the impact of bTB on badger body mass [34], the authors identified that individuals begin to lose mass at advanced stages of infection (respiratory or dissemination stages). To avoid such bTB-related body mass losses influencing the body mass dynamics documented here, all captures of individuals at advanced stages of bTB infection were excluded from the analysis. Age is defined throughout as the number of days elapsed since 20th February in their first year of life, which reflects the mid-February peak in births as exact birth dates cannot be readily determined [35].

### (a) Statistical analysis

All statistical analyses employed linear mixed-effects models using the ‘lmer’ function of lme4 [36] in R v. 2.15.2 [37]. Body mass was used as the response variable in all analyses. Three random intercept terms were included in all models: individual (to account for among-individual variation), social group (to account for heterogeneity in territory quality [38]) and year of capture (to account for temporal environmental heterogeneity [23]). Selective appearance and disappearance (the non-random arrival in, or departure from, the dataset of individuals as age increases) can influence the detection and apparent trajectory of senescence [39]. Selective appearance is not an issue in our analyses as all included individuals were caught in their first year of life. We controlled for selective disappearance by including in all models a linear effect of age at last capture (ALC), defined as the age (in days) at which a badger was last captured [32]. Finally, as terminal effects (a potentially age-independent change in the response term prior to death, due for example to sickness that leads to death) can also complicate the detection and interpretation of age-related patterns [7,40], we included a binary variable reflecting whether or not an individual was in its last year of capture (LYC). To avoid biasing ALC or LYC estimates, all observations from individuals likely to have been alive at the end of the study (those caught in the last 2 years) were excluded from our analyses.

As the analyses used an observational dataset with a large number of candidate explanatory variables and demanded comparisons of nested and non-nested models, we implemented the information theoretic (IT) model selection approach using Akaike's information criterion correcting for small sample size (AICc) [41]. For each of the senescence modelling sections (§2b(i),(ii)), an *a priori* list of candidate models was defined (see below) and then ranked based on AICc. In the interests of parsimony, more complex models were removed from the analysis if a simpler nested version of that model attracted greater support (a lower AICc) [42]. Following such removals, the remaining models with some support (defined as ΔAICc < 6 from the best-supported model) were retained in the top model set [42]. Akaike weights were used to gauge relative support for each model in the top model set and were defined as the likelihood of a given model divided by the total likelihood of all candidate models in the top model set [41]. To calculate the total variance explained by the best-supported model (fixed plus random effects) and the fixed effects alone, we used conditional and marginal *R*^2^ formulations, respectively [43]. Model fit was assessed using standard residual plot techniques.

### (b) Senescence models

#### (i) Sex differences in late-life body mass dynamics

To determine whether the sexes differ in their age-dependent body mass trajectories in late life, a set of 21 candidate models (see the electronic supplementary material, S1) was defined with body mass (kg) as the response term. We used a quadratic age term to capture the curvilinear relationship between chronological age and body mass. Models that included age (in years) as a categorical fixed factor had no support in comparison to quadratic age models and are therefore not discussed further (ΔAICc = + 20.7 relative to the equivalent top quadratic model). In order to preclude the detection of a quadratic age term due to increases in body mass in early life (rather than an accelerating late-life decline), our senescence models only included individuals aged 5 years and over (the timing of the population-level peak in body mass for both sexes; see the electronic supplementary material, S2). This dataset comprises 1241 measures from 297 individuals (107 males, 190 females). We also compared the explanatory power of threshold models and quadratic models to capture age-related changes in body mass (as in [7]; see the electronic supplementary material, S3). Given the outcome, we employed the quadratic approach throughout as it yielded a comparable fit to threshold models in both males (ΔAICc = −0.84 relative to the best-supported threshold model) and females (ΔAICc = +1.29) and better facilitates statistical analysis of the drivers of the magnitude of the sex difference in body mass trajectories (our ultimate goal). In order to test for sex differences in body mass trajectories with age, we therefore assessed the evidence supporting an interaction between sex and both the linear- and squared-age terms that comprise the age-related quadratic. ALC and LYC were included in all models as fixed effects to control for selective disappearance and age-independent terminal changes in body mass, respectively (see above). Following [7], we also allowed for the possibility of a sex-specific terminal decline in body mass, by fitting an interaction between sex and LYC. As previous studies [7,44] have reported effects of month-of-year and current social group size (calculated as the number of unique individuals trapped within a group's territory in a given year) on badger body mass, both were included in all models as covariates.

#### (ii) The influence of early adulthood intra-sexual competition on late-life body mass dynamics

In order to determine whether the strength of intra-sexual competition in early adulthood influences the rate of body mass decline in late life, we defined early adulthood as the first 2 years after sexual maturation (between 365 and 1095 days old; a period in which badgers of both sexes accrue reproductive success [24]). The first 2 years of adulthood were used, rather than the entirety of adulthood, in order to (i) investigate the *downstream* effects of reproductive competition on body mass senescence trajectories, while also controlling statistically for effects of *current* competition on body mass *during* the senescent period and (ii) reduce the colinearity between the density metrics used to describe the competitive environment in early adulthood and the equivalent ‘current’ density metrics calculated during the senescent period.

The modelling process used the best-supported model from §2b(i) (in which body mass was dependent on age and an interaction between age and sex, while controlling for selective disappearance and terminal effects, current social group size and month-of-year) and investigated the effects of three additional predictors that provide proxies for the strength of local intra-sexual reproductive competition in early adulthood: adult male density, adult female density and adult sex ratio (see below for calculation methods). Juveniles (less than 1 year old) were excluded from each as they are unlikely to compete for reproductive opportunities [24]. If allocation to reproductive competition during early adulthood does play a key role in determining senescence rates, then the body mass senescence rates of males are predicted to increase with increasing early adulthood male density and/or increasingly male-biased early adulthood local sex ratios, while those of females may also increase with increasing early adulthood female density. To verify that any detected effect of early adulthood male or female density could not be attributable instead to a correlated effect of total badger density, due for example to generalized foraging competition (as European badgers show no clear sex difference in foraging niche [45]), we also examined the explanatory power of early adulthood total density (local adult male density + adult female density). To allow for the additional possibility that resource competition as a juvenile impacts rates of senescence [31], we also examined the explanatory power of the local population density (local total adult density + local juvenile density) experienced during the juvenile period (0–365 days of age). Finally, having detected apparent downstream effects of early adulthood intra-sexual competition, we investigated whether these could be attributable instead to effects of the (potentially correlated) ‘current’ life competitive environment *during* the senescence period, by contrasting the explanatory power of the early adulthood and ‘current’ life versions of the relevant intra-sexual competition metric.

### (c) Local competition metrics

Local density metrics were estimated by first calculating an annual value for the local density of each class of individuals (adult males, adult females and juveniles) around each badger sett, defined as the total number of unique badgers of each class caught within a 280 m radius of each sett in each year. This search radius provides coverage equal to the mean territory size of a social group across the study period (24.5 ha), constitutes a probable spatial scale for local competition and avoids making assumptions about the relative contributions to local competition of within- or extra-group individuals [26]. Annual trapping effort was standardized in 1986, resulting in high and stable capture probabilities from this year forwards (mean yearly capture probability 1986–2011 = 0.80; s.d. = 0.07; electronic supplementary material, S4). To minimize the impact of annual variation in trapping effort on our density metrics, we therefore restricted our downstream effects analysis to those individuals whose early adulthood period (for which density calculations were required) fell in the years including and following 1986 (902 observations of 229 individuals; 86 males and 143 females).

The annual value for local sex ratio (LSR) for each sett was defined as the ratio of local adult male density to total local adult density (adult male density + adult female density). The proxies for the strength of intra-sexual competition in ‘early adulthood’ (adult male density, adult female density and local sex ratio) for each individual badger were determined by identifying all of the setts at which it was caught during its second and third years of life, and then averaging the relevant annual values of the focal metric for those setts (when a badger was caught at multiple setts during this period, a weighted average was calculated to account for the number of times that it was caught at each set). Likewise, the local population density during the ‘juvenile period’ for each badger was calculated as a weighted average, over all of the setts at which the badger was caught in its first year of life, of the sum of the annual values for local juvenile density and local total adult density. Finally, the ‘current’ life values for each of the proxies for the strength of intra-sexual competition were considered to be the annual value of that metric calculated for the sett at which the badger was caught when the focal body mass measure was taken.

## 3. Results

### (a) Males show a faster rate of body mass senescence than females

**Figure 1.**
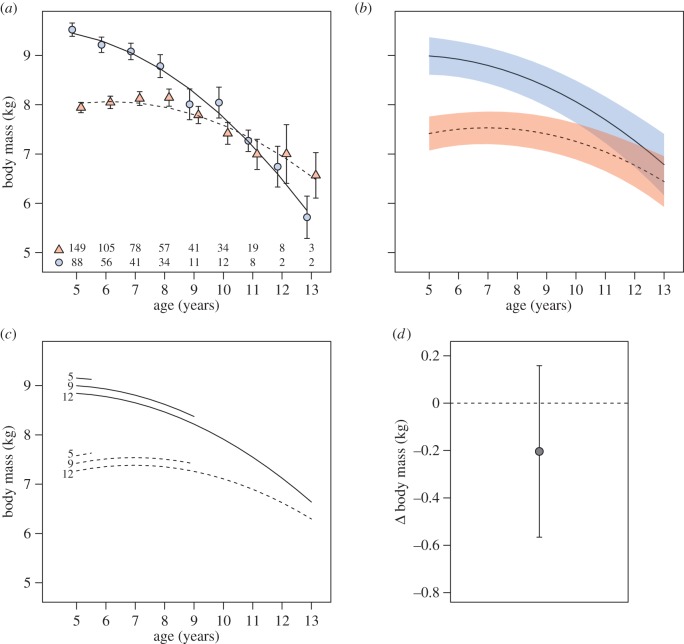
Sex differences in body mass senescence. (*a*) Mean body mass and standard error for males (circles) and females (triangles) for each year of age. Quadratic regression lines were fitted to the means for males (solid) and females (dashed). The numbers within (*a*) present the number of unique individuals of each sex caught within each age class. (*b*) Predicted body masses of males (solid line) and females (dashed line) with age from the top model in table 1. Predictions were for badgers outside of their year of last capture, with ALC and social group size set to their mean values (9.1 and 12.4), and month set to July. The shaded areas present 95% CIs based on fixed effects uncertainty. (*c*) The effect of ALC for males (solid lines) and females (dashed lines) for individuals last caught at ages 5, 9 and 12 years. (*d*) The terminal effect; the predicted change in body mass of individuals in their LYC (whiskers present the 95% CI).

Simple inspection of the raw population-level patterns of body mass change with age after 5 years of age (when peak body mass is attained; see electronic supplementary material, S2), suggests that both sexes show late-life declines in body mass, and that males show steeper late-life declines than females (figure 1*a*). The best-supported linear mixed-model confirms that this decrease in body mass in late life remains after controlling for the effects of social group, year, month of capture, current social group size and both selective disappearance and terminal effects (figure 1*b*; table 1). The interaction between chronological age and sex indicates that the rate of body mass loss with age among males is indeed higher than that among females, and received full support under model selection (table 1; electronic supplementary material, S1). This sex difference cannot be attributed to males simply being larger than females (and so having more mass to lose), as repeating the analysis using standardized body condition in place of body mass yielded similar results (see the electronic supplementary material, S5). The best-supported body mass model (table 1) explained 78.6% of the variation in body mass, of which 42.0% was explained by the fixed effects. The random effects accounted for 36.5% of the model variance, of which 59.6% is accounted for by individual ID, 36.6% by social group and 3.8% by capture year.

**Table 1.** Model selection on the factors affecting body mass during the senescent period (more than or equal to 5 years old) after implementation of a model nesting rule [42]. The grey area denotes the models included in the top set; ✓, terms included in the model; numbers, coefficients; *, interaction between two terms; d.f., degrees of freedom; AW, adjusted weight after removal of more complex models with less support; SGS, current social group size; ALC, age at last capture; LYC, last year of capture. The top model (in bold) was used as the basis for figure 1 and as the base model for the analysis of the downstream effects of competition in early adulthood (presented in table 2). For unabridged model output, see the electronic supplementary material, S1.

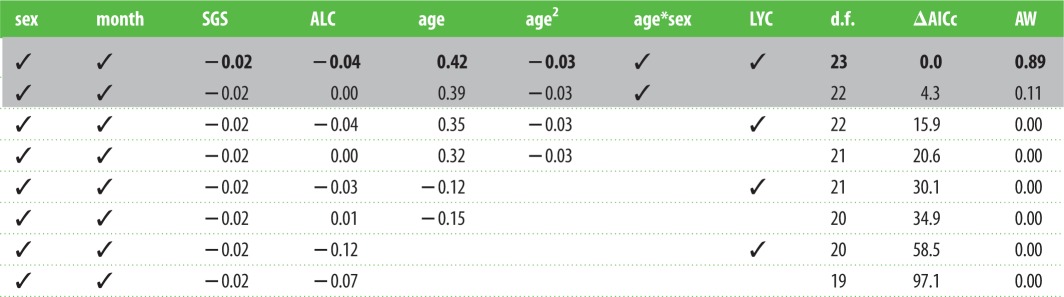

Our models controlled for several additional sources of variation in late-life body mass (table 1). For each year of increase in ALC individuals were on average 39 g lighter (0.5% of average adult body mass), reflecting the selective disappearance of *heavier* individuals from the dataset within increasing age (figure 1*c*; an unusual pattern among mammals, that could reflect a greater likelihood of heavier individuals either dispersing out of the study area or succumbing to road traffic-related mortality [46]). There was also some support for a terminal effect, reflecting an age-independent decrease in body mass of approximately 200 g (2.4% of average adult body mass) in an individual's LYC (figure 1*d*). There was no support for a sex difference in the terminal effect (table 1). Failure to account for these selective disappearance and terminal effects could therefore have falsely exaggerated apparent late-life declines in body mass. Our models also confirmed and controlled for the previously described negative relationship between social group size and body mass (−22 g per group member).

### (b) The sex difference in body mass senescence rate is predicted by the local male density that males experienced during early adulthood

**Figure 2.**
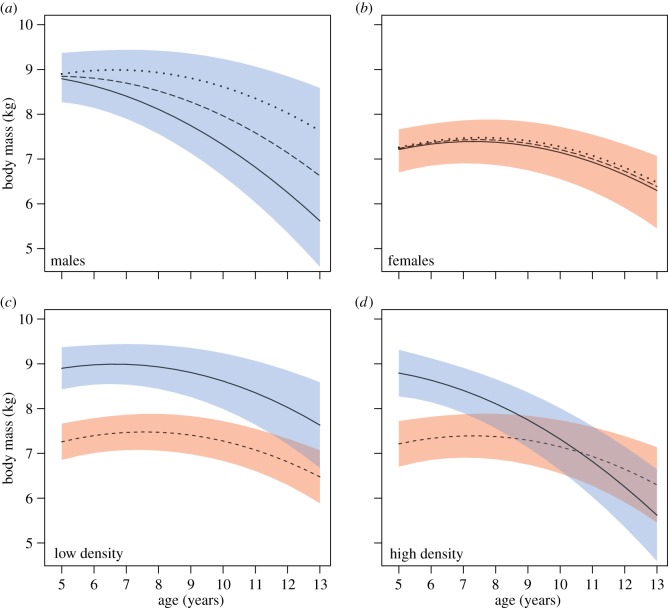
The effect of early adulthood male density on late-life body mass. (*a*,*b*) The predicted relationship between age, the local male density experienced in early adulthood and body mass, from the best-supported model in table 2. Dotted line, low male density (3.7 male per 24.5 ha); dashed line, average male density (5.8); solid line, high male density (7.8). (*c*,*d*) A direct comparison of the sex difference in late-life body mass trajectories of males (solid line) and females (dashed line) under low and high male densities, respectively. Predictions represent badgers outside of their LYC, with ALC and social group size set to their mean values (8.5 and 12.7, respectively), and month set to July. The upper and lower limits of each shaded area represent 95% CI estimates based on fixed effects uncertainty.

The local adult male density that an individual experienced during early adulthood was found to positively predict its rate of late-life decline in body mass for males but not females (the best-supported model contained a three-way interaction between early adulthood male density, age and sex; table 2). Males that experienced higher local male density in the first 2 years of adulthood showed faster rates of body mass senescence than those that experienced lower local male density (figure 2*a*), whereas female body mass senescence rates were unaffected by early adulthood local male density (figure 2*b*). The magnitude of the sex difference in body mass senescence rate is therefore predicted by the intensity of intra-sexual competition experienced by males during early adulthood, with individuals that experienced low adult male densities in early adulthood showing a negligible sex difference in senescence rate (figure 2*c*), while those that experienced high adult male densities in early adulthood showed a marked sex difference in senescence rate (figure 2*d*). Weaker support was also found for a general (i.e. not sex-specific) downstream effect of the total adult density experienced in early adulthood, whereby both males and females that experienced higher total adult densities in early adulthood showed slightly faster rates of body mass decline in late life (table 2; electronic supplementary material, S6).

**Table 2.** Model selection on the competitive metrics hypothesized to influence the rate of body mass senescence. The grey area denotes the models included in the top model set. The first three columns represent terms fitted in addition to the base model (see below), where: *, interactions between terms; +, additive terms; d.f., degrees of freedom; AW, adjust weight after removal of more complex models with less support; EA, early adulthood; LSR, local sex ratio; JP density, local population density during the juvenile period (0–365 days old). The base model was defined as: body mass ∼ month + ALC + SGS + LYC + age*sex + age2 + (1|ID) + (1|year) + (1|social group); the top model from table 1.

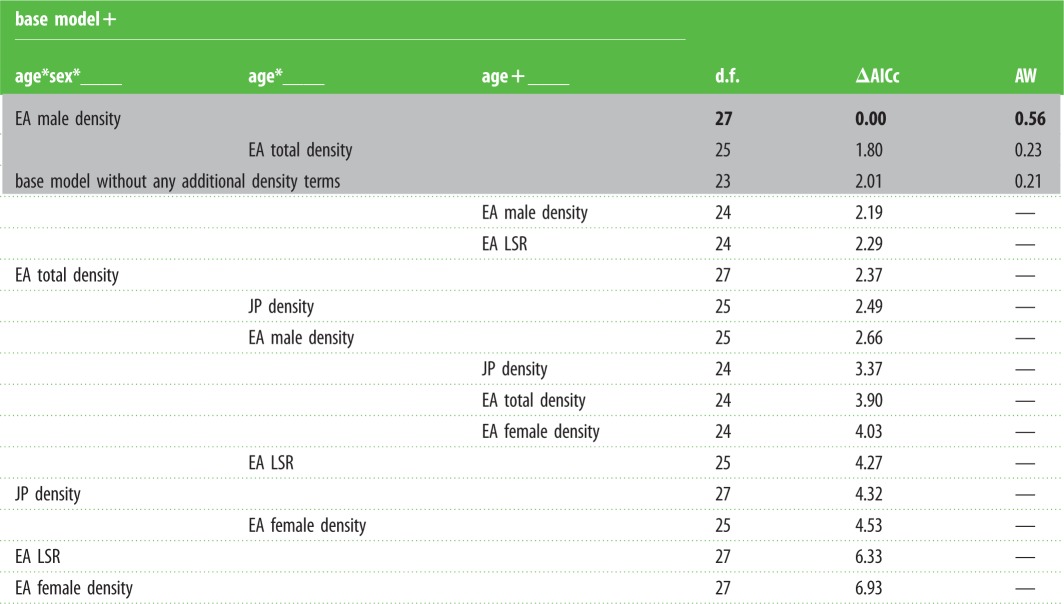

Direct comparison of the explanatory power of the three proxies for the strength of intra-sexual reproductive competition in early adulthood indicated that early adulthood male density had the strongest support as a predictor, while early adulthood female density and local sex ratio had none (table 2). The weight of evidence also suggests that the statistical support for a sex-specific downstream effect of male density in early adulthood is unlikely to be a simple by-product of a correlation between male density and total adult density in early adulthood, as there was no support for a sex-specific downstream effect of total adult density (table 2). Likewise, there was no support for a downstream effect (sex-specific or otherwise) of the local population density experienced during the juvenile period on body mass later in life (table 2). Finally, the statistical support for a downstream effect of male density in early adulthood also cannot be readily attributed to a correlated direct effect of the ‘current’ male density experienced *during* the senescent period, as comparing the explanatory power of the two predictors yielded strong support for a role for early adulthood male density (electronic supplementary material, S7).

## 4. Discussion

Our longitudinal analyses reveal a clear sex difference in the rate of late-life decline in body mass in European badgers; males show faster rates of decline than females. As body mass is positively associated with reproductive success [21,22] and survival [23] in this species, steeper body mass senescence among males than females provides one likely explanation for the steeper late-life declines in both reproductive success and survival recently documented among male European badgers [24,25]. As overt intra-sexual reproductive competition appears to be more intense among male European badgers than females, the observed sex difference in late-life body mass dynamics is consistent with a role for intra-sexual reproductive competition in generating sex differences in senescence rates, and echoes previous reports of higher senescence rates among males in polygynous species [3,5,16]. Uniquely, however, our findings also suggest that individual variation in the intensity of intra-sexual competition experienced in early adulthood may generate downstream variation in body mass senescence rates among males but not females, and hence influence the extent of the sex difference observed in body mass senescence rates. As such, our findings provide rare support for the view that sex differences in senescence are not simply an evolved consequence of sex differences in mortality rates, but may also arise in part from downstream effects of intra-sexual competition experienced during the lifetime.

Multiple lines of evidence suggest that intra-sexual reproductive competition is more intense among male European badgers than females; males contest matings within and between groups, show higher rates of bite-wounding and mortality than females, and the species shows male-biased sexual size dimorphism [29,30]. As such, the observed sex difference in mean body mass senescence rates is compatible with both prevailing theories that invoke a key role for intra-sexual reproductive competition in generating sex differences in senescence. First, classical evolutionary theory predicts that steeper senescent declines should arise among males in polygynous species because the higher male mortality rates that often accompany polygyny are expected to differentially weaken selection among males against deleterious mutations or antagonistically pleiotropic genes acting in late life [10]. Second, mechanistic approaches also predict steeper senescent declines in the sex that experiences stronger intra-sexual reproductive competition, owing to somatic maintenance costs arising from their differential investment in competitive morphologies and behaviour [11,14].

However, our results also suggest that both the individual variation and sex difference in late-life body mass dynamics in this population arise in part from downstream effects of the intensity of intra-sexual competition experienced in early adulthood. A direct comparison of the sexes revealed support for a positive effect of early adulthood local male density on the rate of body mass senescence in males but not females. This apparent downstream effect of the intensity of intra-sexual competition experienced *within* a male's lifetime is important, as it is not predicted by classical evolutionary approaches that consider sex differences in senescence solely a product of sex differences in mean mortality schedules [16]. Such downstream effects of intra-sexual competition are, however, predicted if sex differences in senescence are envisaged to arise from a trade-off between allocation to reproductive competition and somatic maintenance [11,14]. Indeed, if such a trade-off in males played a central role in generating sex differences in senescence rate, male badgers experiencing low levels of intra-sexual competition in early adulthood would be predicted to show senescence rates akin to the population average for females, which is what we found (cf. figure 2*c,d*). As such, our findings provide support for the hypothesis that sex differences in senescence rate arise in part from somatic maintenance costs entailed in intra-sexual competition.

We were able to evaluate several potential alternative explanations for the statistical evidence that male density in early adulthood predicts the rate of late-life decline in body mass among males. First, this finding cannot be readily attributed instead to a sex-specific downstream effect of the *total* adult density experienced in early adulthood, as our analysis revealed no support for such a model. Our analysis did reveal weak support for a general (not sex-specific) downstream effect of the total adult density experienced in early adulthood on the senescence rates of all individuals (potentially attributable to density-dependent somatic maintenance costs [31]), but it seems unlikely that such an effect alone could account for our findings as the model reflecting a sex-specific downstream effect of male density in early adulthood attracted stronger support (table 2). Indeed, extending our statistical analysis suggests that the downstream effect of total density may be acting in tandem with the sex-specific downstream effect of male density (the two need not be mutually exclusive); an aggregate model including both downstream effects (a possibility untested in table 2) attracts stronger support than the current top model (ΔAIC = −2.93) while leaving the parameter estimates for both downstream effects qualitatively unchanged (electronic supplementary material, S6). The sex-specific effect of early adulthood male density also cannot be readily attributed instead to a direct effect of the male density experienced *during* the senescence period (potentially correlated with male density during early adulthood), as competing these models revealed stronger support for an effect of *early adulthood* male density (electronic supplementary material, S7). Likewise, all of our models control for the previously documented effects of social group size on a badger's current body mass [44,47]. As our models also control for both selective disappearance and terminal effects, it is also unlikely that either process is leading here to an overestimation of late-life declines in body mass.

That the local adult sex ratio experienced in early adulthood had no detectable effect on late-life body mass declines in either sex is not unexpected, as the strength of male–male competition for matings at any one time may be largely unrelated to the local density of females for two reasons. First, as females can exhibit asynchronous oestrus over a long reproductive season (with births seasonally synchronized by delayed implantation) and second, as only a subset of adult females within a group successfully breed [21,28].

Given recent interest in the evolutionary consequences of female–female competition [48], it is notable that we found no detectable effect of early adulthood female density on the late-life body mass dynamics of females. Lower frequency of bite-wounding and lower mortality among females, coupled with male-biased sexual dimorphism, are consistent with intra-sexual competition being less intense among females, and this alone could explain the lack of an evident downstream effect of early adulthood female density. Such patterns also accord with recent suggestions that females may be more likely than males to resolve conflict without escalated physical contests [49]; a pattern that could itself weaken intra-sexual selection among females for investment in competitive traits [50] and, by extension, any associated costs of doing so for late-life performance.

Together, our findings provide rare support for the view that sex differences in senescence rates arise in part from downstream effects of trade-offs between allocation to intra-sexual reproductive competition and somatic maintenance [11,14], and highlight that such trade-offs may shape both inter-individual differences and sex differences in senescence rates in wild populations. That trade-offs of this kind might also account for the sex differences in senescence documented in other polygynous species [4,5,16], as well as interspecific variation in their magnitudes [3], highlights the need for caution when attributing such patterns to evolutionary responses to sex differences in mean mortality rates. Our findings suggest that attempts to understand the origins of sex differences in senescence may be well served by focusing on the behavioural and molecular mechanisms that may generate trade-offs between exposure to intra-sexual competition during the lifetime and aspects of late-life performance.

## Supplementary Material

Electronic Supplementary Material

## Data accessibility

Body mass dataset available at Dryad: http://dx.doi.org/10.5061/dryad.607th.
