# Supplementary material for: Sex differences in senescence: the role of intra-sexual competition in early adulthood
Source: Proc Biol Sci. 2015 Jul 22;282(1811):20151086. doi: 10.1098/rspb.2015.1086 (PMC4528560; doi:10.1098/rspb.2015.1086)
Supplement: Electronic Supplementary Material [file rspb20151086supp1.pdf]

Electronic Supplementary Materials

Int	Sex	Month	SGS	ALC	Age	Age^2	Age*Sex	Age^2*Sex	LYC	Sex*LYC	Age_{cat}	Age_{cat}*Sex	df	logLik	ΔAICc	AW	RET
8.67	✓	✓	-0.02	-0.04	0.42	-0.03	✓		✓				23	-1791	0.00	0.89	✓
8.67	✓	✓	-0.02	-0.04	0.42	-0.03	✓		✓	✓			24	-1791	1.65	0.00	
8.76	✓	✓	-0.02	-0.04	0.39	-0.03	✓	✓	✓				24	-1791	2.01	0.00	
8.76	✓	✓	-0.02	-0.04	0.39	-0.03	✓	✓	✓	✓			25	-1791	3.65	0.00	
8.43	✓	✓	-0.02	0.00	0.39	-0.03	✓						22	-1794	4.25	0.11	✓
8.48	✓	✓	-0.02	0.00	0.38	-0.03	✓	✓					23	-1794	6.30	0.00	
9.00	✓	✓	-0.02	-0.04	0.35	-0.03			✓				22	-1800	15.89	0.00	
8.97	✓	✓	-0.02	-0.04	0.35	-0.03			✓	✓			23	-1799	16.60	0.00	
8.76	✓	✓	-0.02	0.00	0.32	-0.03							21	-1804	20.62	0.00	
9.98	✓	✓	-0.02	-0.04					✓		✓	✓	36	-1788	20.71	0.00	
9.99	✓	✓	-0.02	-0.04					✓	✓	✓	✓	37	-1788	22.71	0.00	
9.98	✓	✓	-0.02	-0.04					✓		✓		28	-1798	23.79	0.00	
9.62	✓	✓	-0.02	0.00							✓	✓	35	-1791	23.84	0.00	
9.96	✓	✓	-0.02	-0.03					✓	✓	✓		29	-1797	24.65	0.00	
9.60	✓	✓	-0.02	0.00							✓		27	-1801	27.21	0.00	
10.72	✓	✓	-0.02	-0.03	-0.12				✓				21	-1808	30.06	0.00	
10.72	✓	✓	-0.02	-0.03	-0.12				✓	✓			22	-1808	31.27	0.00	
10.49	✓	✓	-0.02	0.01	-0.15								20	-1812	34.90	0.00	
10.68	✓	✓	-0.02	-0.12					✓				20	-1824	58.50	0.00	
10.68	✓	✓	-0.02	-0.12					✓	✓			21	-1823	60.29	0.00	
9.97	✓	✓	-0.02	-0.07									19	-1844	97.08	0.00	

Table S1. The unabridged output table for model selection on the factors affecting body mass during the senescent period (≥ 5 years old). The abridged version is presented as Table 1 in the main manuscript. The final column indicates those models that were retained after the application

of the nesting rule (Richards *et al.* 2011; only these models appear in Table 1 in the main manuscript), whereby models are removed if they are more complex versions of nested (simpler) models that attracted stronger support. The grey area denotes the models included in the top set, ✓ = terms included in the model, * = interaction between two terms, Int = intercept, SGS = Social Group Size, ALC = Age at Last Capture, Age = Age in days, Age_{cat} = Age coded categorically (years), LYC = Last Year of Capture, df = degrees of freedom, AICc = Akaike's Information Criterion corrected for small sample size, ΔAICc = change in AICc relative to best supported model, AW = adjusted weight after removal of more complex models with less support.

Electronic Supplementary Material S2

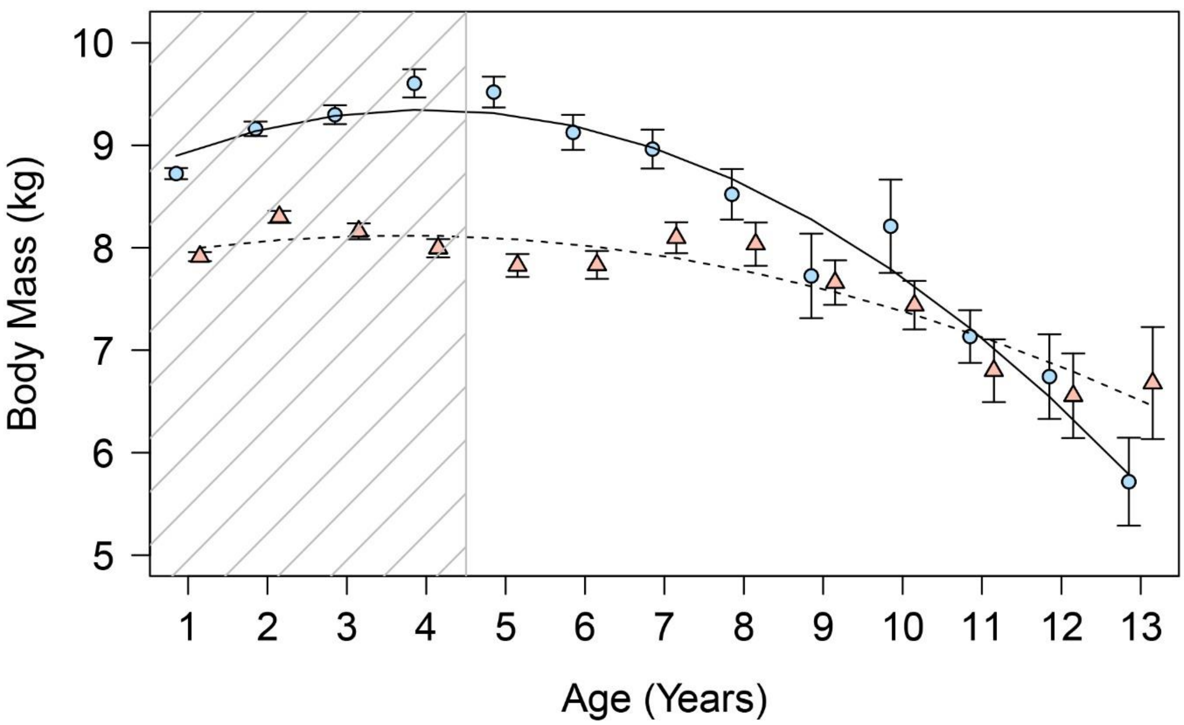

Figure S1. Removal of individuals prior to peak body mass. Observations prior to five years of age (grey hatched area) where removed from all body mass analyses to ensure that the age-related body mass dynamics prior to the attainment of peak body mass (which likely relate more to growth than senescence) did not influence our statistical conclusions regarding the age-related body mass dynamics in later life. The mean body masses and standard errors for males (circles) and females (triangles) for each year of age, for all individuals aged one year and over. The lines represent quadratic regression lines fitted to the means for males (solid line) and females (dashed line). The hatched grey area represents the age-classes excluded from the senescence analysis dataset.

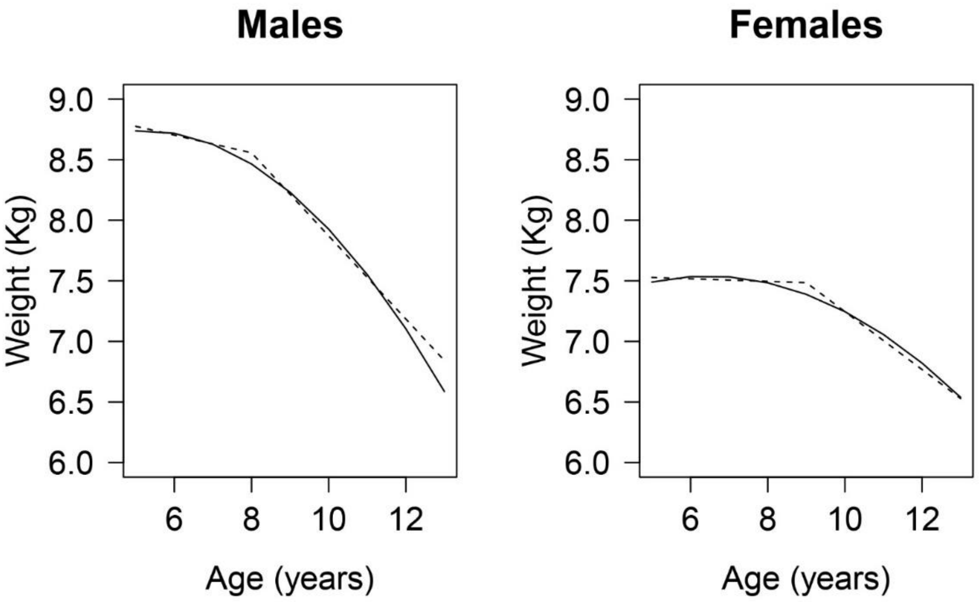

Figure S2. Comparison of threshold and quadratic models of late-life body mass. We compared a range of single threshold (between 6 and 12 years inclusive) and double threshold (first threshold 6-10 years inclusive; second threshold 8-12 years inclusive) piecewise regression models to the best supported quadratic age function (Age*Sex + Age^2; the top model in Table 1) for males and female respectively. The best supported threshold model for males (single break point at 8 years; dashed line) received less support than the quadratic age model (ΔAICc = +0.84 relative to the quadratic; solid line), whereas the best supported threshold model for females (single break point at 9 years; dashed line) received more support (ΔAICc = -1.29 relative to the quadratic; solid line).

Assessing the effect of variation in recapture rates. The simple local density metric used here (based on the number of unique individuals caught within a 280m radius of a sett in each calendar year) could be influenced by variation in recapture probabilities both through time and between the sexes. To determine the extent to which this is the case we fitted a series of Cormack-Jolly-Seber models to assess the evidence for *i)* year-year variation in the recapture rate, *ii)* sex-specific recapture rate and *iii)* an interaction between year and sex (which could lead to temporal bias our estimates of local sex ratio). Candidate models were assessed using Akaike's Information Criteria (AIC) adjusted for overdispersion (QAIC), whereby 'better' candidate models are indicated by lower AIC values. We tested for overdispersion of models using the 'program RELEASE' method as implemented in the program MARK, and applied an overdispersion estimate of 1.73 to all of the results. As in Graham *et al.* (2013) we applied time invariant sex-specific survival parameter. We found support for year-year variation and sex-specific recapture probabilities (males were more likely to be recaptured than females). As adjusting the simple local density metrics to account for variation in recapture probabilities yielded results which were qualitatively unchanged we only report the results from using simple local density in the manuscript.

Recapture	Survival	QAIC	ΔQAIC	Num. of Parameters	QDeviance
**Year + Sex**	**Sex**	**6850.7**	**0.00**	**28**	**1621.0**
Sex	Sex	6853.4	2.74	4	1672.0
Year	Sex	6854.1	3.40	27	1626.0
-	Sex	6856.8	6.16	3	1677.0
Year * Sex	Sex	6891.9	41.20	52	1613.6

Table S2. Summary of the model selection on factors influencing recapture probability. Where: Recapture = the terms included in estimation of recapture probability; Survival = terms included in the estimation of survival probability; QAIC = Quasi-Akaike's Information Criteria after over dispersion correction; ΔQAIC = change in QAIC in comparison to the best

supported model; QDeviance = Quasi-deviance for each model. The model in bold denotes the best supported model.

1.

Graham J, Smith GC, Delahay RJ *et al.* (2013) Multi-state modelling reveals sex-dependent transmission, progression and severity of tuberculosis in wild badgers. *Epidemiology and Infection*, **141**, 1429–1436.

Repeating analysis with Body condition. In order to address the possibility that males show steeper late-life declines in body mass simply because they are larger individuals with more body mass to lose, we repeated our analysis from Section A in the main paper using a metric of standardised body condition, the Scaled Mass Index (SMI; Peig & Green 2010) in place of body mass. The SMI accounts for the sexual dimorphism in body size by scaling the body masses of all individuals (of differing body sizes) to the value expected for a single standardised body length, utilising the species-specific allometric scaling relationship between body length and body mass (Peig & Green 2010). Accordingly, the sex difference in mean body mass in European badgers in our data set (Males: 8.85kg, Females: 7.77kg) is no longer apparent in SMI (Males: 8.74kg, Females: 8.67kg) and, likewise, the higher variance in body mass among males due most likely to their larger size (Males: 2.95, Females: 2.55) is no longer apparent in SMI, indeed the reverse is true (Males: 2.04, Females: 2.72). Using SMI, body mass losses are therefore weighted against an individual's size, leaving a larger absolute change in body mass required in longer individuals than shorter individuals to bring about a comparable change in SMI. The SMI has been found to capture variation in fat and protein reserves more effectively than traditional residual body condition indices (Peig & Green 2009), and thus makes it an appropriate index for use in senescence studies. Body length measurements (the distance in centimetres from the tip of the nose to the distal point of the last caudal vertebra) were only available for badger captures from 1997 onwards, which substantially reduced the data set of captures of known-age individuals in late-life to just 536 observations (a reduction of 57%) when using SMI as the response variable in place of body mass. The 'scaling component' was estimated through standardised major axis regression of ln(body mass) on ln(body length) to be 4.5 (following Peig & Green 2010).

Repeating the analysis in Section A of the main manuscript using SMI as the response term in place of body mass confirmed support for a sex difference in the rate of standardised body condition (SMI) loss with age in late-life, whereby the SMIs of males declined at a faster rate than those of females (Figure S2a), again while controlling for terminal effects, selective disappearance effects, current social group size, month of capture and the random effects (Table S3). Consistent with the results of the body mass modelling presented in the main text, the SMI analysis suggests that SMI decreases with increasing age at last capture (a selective disappearance effect; Figure S2b) and in the last year of capture (a terminal effect; Figure S2c). Whilst the best-supported model contained the age*sex interaction, two models within the ΔAICc < 6 top model set did not (Table S3), which likely reflects the marked reduction in the available sample size when using SMI in place of body mass .

1.

Peig, J. & Green, A.J. (2009). New perspectives for estimating body condition from mass/length data: the scaled mass index as an alternative method. *Oikos*, 118, 1883–1891.

2.

Peig, J. & Green, A.J. (2010). The paradigm of body condition: a critical reappraisal of current methods based on mass and length. *Funct. Ecol.*, 24, 1323–1332.

3.

Richards, S., Whittingham, M. & Stephens, P. (2011). Model selection and model averaging in behavioural ecology: the utility of the IT-AIC framework. *Behav. Ecol. Sociobiol.*, 65, 77–89.

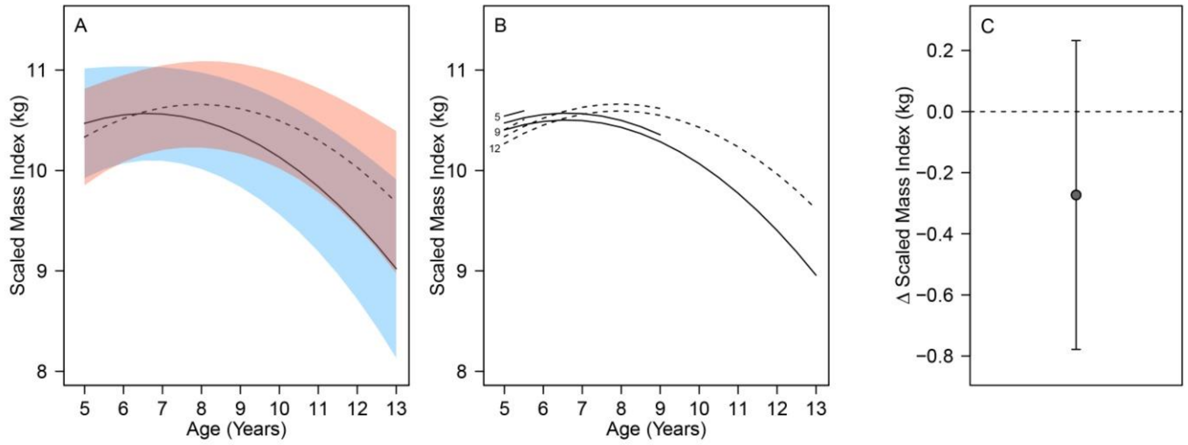

Figure S3. **Model output from body condition analyses.** (**a**) presents predicted SMI of males (blue/solid line) and females (red/dashed line) with advancing age from the top model in Table S3. Predictions were made for badgers outside of their year of last capture, with ALC and social group size set to their mean values (9.2 and 12 respectively), and month set to July. The shaded areas represent 95% confidence intervals based on fixed effects uncertainty. (**b**) presents the effect of age at last capture (ALC) for males (solid lines) and females (dashed lines) for individuals last caught at ages 5, 9 and 12 years. (**c**) presents the terminal effect; the predicted change in SMI of individuals in their last year of capture (whiskers present the 95% confidence interval).

Int	Sex	Month	SGS	ALC	Age	Age^2	Age*Sex	Age^2*Sex	LYC	Sex*LYC	df	ΔAICc	AW	RET
8.94	✓	✓	-0.08	-0.02	0.60	-0.04	✓		✓		21	0.00	0.55	✓
8.92	✓	✓	-0.08	-0.02	0.61	-0.04	✓		✓	✓	22	0.78	0.00	
9.15	✓	✓	-0.08	-0.03	0.58	-0.04			✓		20	1.19	0.30	✓
9.11	✓	✓	-0.08	-0.02	0.56	-0.04	✓	✓	✓		22	2.01	0.00	
9.12	✓	✓	-0.08	-0.02	0.55	-0.03	✓	✓	✓	✓	23	2.72	0.00	
9.17	✓	✓	-0.08	-0.03	0.58	-0.04			✓	✓	21	3.13	0.00	
8.48	✓	✓	-0.08	0.04	0.58	-0.04	✓				20	3.22	0.11	✓
8.66	✓	✓	-0.08	0.04	0.54	-0.04	✓	✓			21	5.21	0.00	
8.68	✓	✓	-0.08	0.04	0.56	-0.04					19	5.26	0.04	✓
11.74	✓	✓	-0.08	-0.08					✓		18	13.52	0.00	
11.71	✓	✓	-0.08	-0.03	-0.06				✓		19	13.71	0.00	
11.75	✓	✓	-0.08	-0.08					✓	✓	19	15.13	0.00	
11.71	✓	✓	-0.08	-0.04	-0.05				✓	✓	20	15.53	0.00	
11.32	✓	✓	-0.08	0.04	-0.12						18	19.09	0.00	
11.09	✓	✓	-0.07	-0.05							17	28.88	0.00	

Table S3. **Model selection on the factors affecting body condition (SMI) during the senescent period (≥ 5 years old).** The final column indicates those models that were retained after the application of the nesting rule (Richards *et al.* 2011), whereby models are removed if they are more complex versions of nested (simpler) models that attracted stronger support. The grey area denotes the models included in the top set, ✓ = categorical terms included in the model, * = interaction between two terms, Int = intercept, SGS = Social Group Size, ALC = Age at Last Capture, Age = Age in days, LYC = Last Year of Capture, df = degrees of freedom, AICc = Akaike's Information Criterion corrected for small sample size, ΔAICc = change in AICc relative to best supported model, AW = adjusted weight after removal of more complex models with less support.

Electronic Supplementary Material S6

The non-sex-specific downstream effect of total density in early adulthood on late life declines in body mass. Individuals experiencing high early adulthood population density show faster late life declines in body mass (Figure S4), which concurs with the density effect found in (Nussey *et al.* 2007). Extending our statistical analyses suggests that this downstream effect of total density is acting in addition to the sex-specific downstream effect of male density, as the inclusion of *both* downstream effects in a single model (a model that Table 2 does not test: Body mass ~ Month + ALC + Social Group Size + LYC **+ Age*Sex*Early Adulthood Male Density + Age*Early Adulthood Total Density** + Age^2 + 1|ID + 1|Year + 1|Social Group) yielded a model with stronger support than any of our existing models (i.e. a better AIC than the best supported model in Table 2; ΔAIC= -2.96; adjusted model weight 0.71) with parameter estimates for the downstream effects of early life male density and early life total density qualitatively unchanged.

1. Nussey, D.H., Kruuk, L.E.B., Morris, A. & Clutton-Brock, T.H. (2007) Environmental conditions in early life influence ageing rates in a wild population of red deer. *Curr. Biol.*, 17, R1000–R1001.

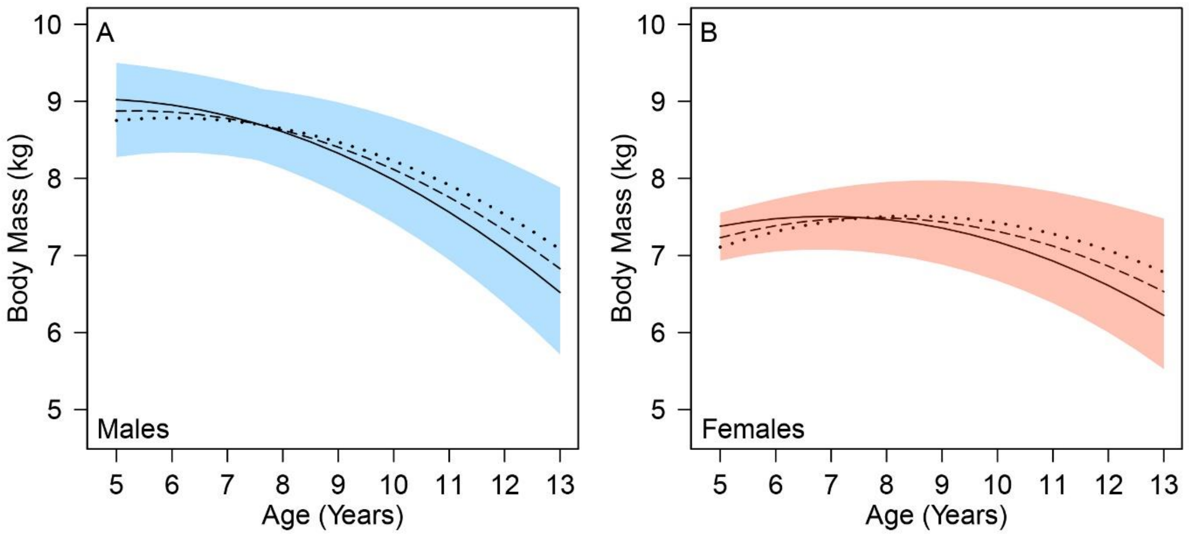

Figure S4. The downstream effect of early adulthood total density on the late-life body masses of (a) males and (b) females (though this was *not* a sex-specific effect). The panels present the predicted relationship between age, the total adult density experienced in early adulthood and body mass, from the second ranked model in Table 2. Dotted lines = low total density (12.5 individuals per 24.5 hectares); dashed lines = average total density (16.2); solid lines = high total density (22.2). Predictions represent badgers outside of their year of last capture, with age at last capture and social group size set to their mean values, and month set to July. The upper and lower limits of each shaded area represent 95% confidence interval estimates based on fixed effects uncertainty.

Electronic Supplementary Material S7

	Base model +				
Age^2*Sex*____	Age^2*____	Age^2+____	df	ΔAICc	AW
**EA Male Density**			**27**	**0.00**	**0.68**
		EA Male Density	24	2.19	0.23
	EA Male Density		25	2.66	-
		Current Male Density	24	4.03	0.09
	Current Male Density		25	5.33	-
Current Male Density			27	7.78	-

Table S4. Model selection directly comparing the explanatory power of interactions between age and male density experienced in early adulthood ('EA Male Density') or male density experienced during the senescent period ('Current Male Density'). The grey area denotes the models included in the top model set. The first three columns represent terms in addition to the base model (see below), where: * = interactions between terms, + = additive terms, df = degrees of freedom, AW = adjusted weight after removal of more complex models with less support. The base model was defined as: Body mass ~ Month + ALC + Social Group Size + LYC + Age*Sex + Age^2 + (1|ID) + (1|Year) + (1|Social Group); the top model from Table 1.
